# Expansion of Protein Domain Repeats

**DOI:** 10.1371/journal.pcbi.0020114

**Published:** 2006-08-25

**Authors:** Åsa K Björklund, Diana Ekman, Arne Elofsson

**Affiliations:** Stockholm Bioinformatics Center, Center for Biomembrane Research, Stockholm University, Stockholm, Sweden; University of California San Diego, United States of America

## Abstract

Many proteins, especially in eukaryotes, contain tandem repeats of several domains from the same family. These repeats have a variety of binding properties and are involved in protein–protein interactions as well as binding to other ligands such as DNA and RNA. The rapid expansion of protein domain repeats is assumed to have evolved through internal tandem duplications. However, the exact mechanisms behind these tandem duplications are not well-understood. Here, we have studied the evolution, function, protein structure, gene structure, and phylogenetic distribution of domain repeats. For this purpose we have assigned Pfam-A domain families to 24 proteomes with more sensitive domain assignments in the repeat regions. These assignments confirmed previous findings that eukaryotes, and in particular vertebrates, contain a much higher fraction of proteins with repeats compared with prokaryotes. The internal sequence similarity in each protein revealed that the domain repeats are often expanded through duplications of several domains at a time, while the duplication of one domain is less common. Many of the repeats appear to have been duplicated in the middle of the repeat region. This is in strong contrast to the evolution of other proteins that mainly works through additions of single domains at either terminus. Further, we found that some domain families show distinct duplication patterns, e.g., nebulin domains have mainly been expanded with a unit of seven domains at a time, while duplications of other domain families involve varying numbers of domains. Finally, no common mechanism for the expansion of all repeats could be detected. We found that the duplication patterns show no dependence on the size of the domains. Further, repeat expansion in some families can possibly be explained by shuffling of exons. However, exon shuffling could not have created all repeats.

## Synopsis

The building blocks that create proteins are called domains, and domains are often combined to create multidomain proteins. In many vertebrate proteins, repeats with several adjacent domains from the same family can be found. The authors have investigated how these repeats may have evolved. It is believed that the repeats are created through internal duplications where the duplicated region is inserted next to its origin. Therefore, the pairwise sequence similarity between all repeated domains in a protein was used to identify recent duplications, and a method based on autocorrelation vectors was employed to distinguish patterns of duplication. The authors found that repeat regions are often created from the duplication of several domains at a time while duplication of one domain is less common. Further, the internal duplications often occur in the middle of the repeats. This is in contrast to the evolution of nonrepeating, multidomain proteins, which are thought to evolve by the addition of a single domain at the N-termini or C-termini. A preference for duplication of a certain number of domains was found for some of the domain families. Finally, the authors discuss some of the possible mechanisms for repeat expansion. However, the exact mechanism remains to be discovered.

## Introduction

Proteins are composed of domains, recurrent protein fragments with distinct structure, function, and evolutionary history. Protein domains may occur alone, but are more frequently found in combination with other domains in multidomain proteins. While the creation of new multidomain architectures through shuffling of protein domains has been studied extensively during the last few years [1–4], one type of domain recombination has often been ignored: the creation of domain repeats. Domain repeats contain two or more domains from the same domain family in tandem. Large repeats with more then ten domains in tandem are common in eukaryotes.

Repeating domains are often short, such as the leucine rich repeat (LRR) family with a repeating unit of 30 residues. Some repeated domain families are mainly found in repeats, e.g., LRR and C2H2 zinc fingers, while other families are also frequently found as a single unit. The repeats may form regular structures, such as antiparallel β-sheets or solenoids, while others form filaments or are only structured upon binding to their ligands [5]. Some examples of repeats in protein structures can be found in the Propeat database (http://gln.ibms.sinica.edu.tw/product/repeat/). Single amino acids or short peptide motifs may be repeated in proteins, too. However, in this study we have focused on larger repeating units, domains. Therefore, when repeats are mentioned in this text, it refers to repeats of protein domains.

Domain repeats are often involved in interactions with proteins or other ligands such as DNA or RNA. Even if the repeated domains have a well-defined and conserved structure, the sequence conservation is often low, with only a few conserved residues required for the correct fold. Their variable sequences and the variation in number of domains provide flexible binding to multiple binding partners. Hence, repeats are found in proteins with highly diverse functions such as the tetratrico peptide repeats (TPR) that are involved in cell-cycle regulation, transcriptional regulation, protein transport, and assisting protein folding [6]. In addition, the flexible binding properties and sequence variability of repeats have been exploited to create high affinity binders as an alternative to antibodies [7].

The domain repeats are found in all kingdoms of life, and long repeats, containing several domains in tandem, have been observed to be particularly common in multicellular species [1,8]. Repeats have been proposed to provide the eukaryotes with an extra source of variability to compensate for low generation rates [9]. One such example is the LRRs in plant defense systems that enable plants to adapt to new pathogens [10].

Domain repeats are thought to arise via tandem duplications within a gene [5], where a segment is duplicated and the copy is inserted next to its origin. However, the exact mechanism behind this phenomenon is not fully understood. Nonhomologous recombination in intron regions, i.e., exon shuffling, may be responsible for internal duplications in repeats, and this issue has been addressed in this study. Another possible explanation is DNA slippage, due to the formation of DNA hairpins, which is common in the creation of nucleotide repeats and short protein repeats [11]. However, Marcotte and coworkers have shown that protein repeats are more likely created from recombination than by DNA slippage since the repeat expansion shows weak dependence on repeat length [9].

In addition to internal duplications, frequent duplications of repeat-containing genes have occurred in the mammalian genomes [12]. This can, in part, explain their abundance in higher eukaryotes. In addition, variation in number of repeats between orthologous genes indicates that the loss/gain of domains in repeats is frequent in evolution [12]. Interestingly, the rapid expansion of repeats in eukaryotes could partly be explained by tandem duplication of units containing several repeated domains [12–15]. In this study, we aim to investigate how frequent duplications of multiple domains are. Further, the number of domains that is duplicated is compared among the different domain families. Domains as defined by the Pfam-A database [16] were detected using HMM-alignments. The coverage was increased with relaxed detection criteria for domains in repeated regions of the proteins. In addition to investigation of duplication sizes, the domain assignments have been used to study the distribution of repeats and repeated domain families in the three kingdoms of life, the position of repeat expansion, and the location of exon boundaries in repeats.

## Results/Discussion

### Repeats are Frequent in Vertebrates

**Figure 1:**
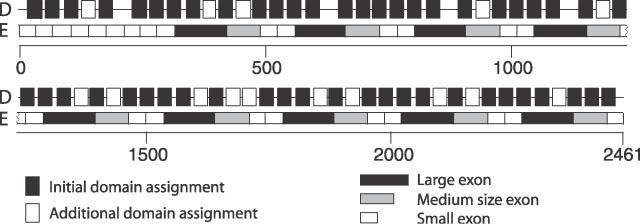
Domain Assignments and Exon Structure for the Chicken Nebulin Protein ENSGALP00000020382 The initial domain assignments (D) using an E-value cutoff at 0.1 detected 51 nebulin domains. With a less strict cutoff, we were able to assign 15 additional domains. Still, there are four gaps (regions with no domain assignment), which are likely to contain domains that cannot be detected with the current HMMs. Below the domain assignments, the exon structure (E) is seen, with a box for each of the 44 exons, where it is evident that a block of four exons (a long one in black, two short ones in white, and one intermediate size in gray) correspond to a block of seven domains even if the exon borders all are found within the domains.

**Figure 2:**
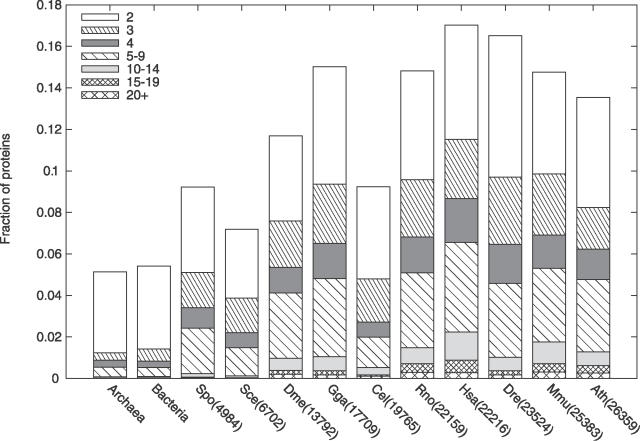
Fraction of Proteins That Contain a Domain Repeat in Archaea, Bacteria, Yeast, and the Eight Multicellular Eukaryotes (Sorted by Number of Proteins) The different patterns indicate the length of the repeat, i.e., whether it contains 2, 3, 4 domains, etc. The eukaryotic species are labeled with the abbreviations of species names such as Hsa for Homo sapiens followed by the number of proteins in each proteome. For a list of all species in this study, see Materials and Methods.

It has been demonstrated that protein domain repeats are particularly abundant in multicellular organisms [1,8]. However, multicellularity does not seem to be the sole determinant for having many repeats. Using extended domain assignments (Figure 1), the fractions of the different proteomes that consist of proteins with repeats were compared. As has been shown for other types of protein repeats [17], more complex organisms seem to require more domain repeats. Consequently, the fraction of proteins with repeats is higher for species with large proteomes, especially when repeats of three or more domains are considered (Figure 2, Table 1). Plants and vertebrates, particularly humans, contain many proteins with domain repeats (Figure 2). However, the eukaryotes Arabidopsis thaliana and especially Caenorhabditis elegans have fewer repeats than expected from their proteome size. Actually, the worm and the two yeast species have a similar fraction of proteins with repeats, hence multicellular organisms are not always distinguished by more repeats than unicellular ones. In addition, some prokaryotes, such as Escherichia coli and *Pseudomonas aeruginosa,* with similar proteome size as yeast, contain very few proteins with repeats. Thus, having many repeats is a feature of eukaryotes rather than of multicellular species.

**Table 1:**
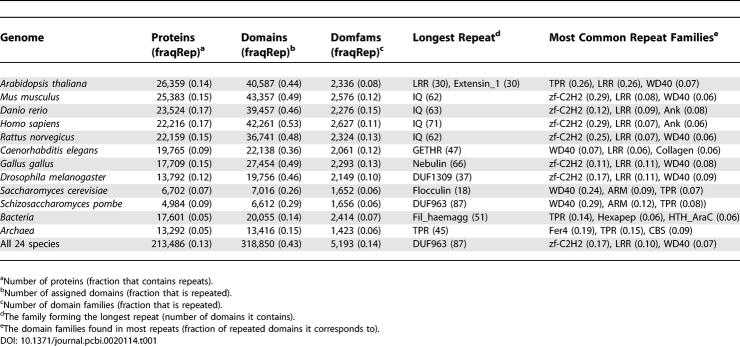
Summary of Repeat Distribution in the Different Species

As many proteins with repeats of more than two domains are found in vertebrates, they should provide functions that are required in complex organisms. Consistently, the proteins with repeats mainly have important binding functions in protein–protein interactions and complex assembly as demonstrated for the largest domain families in Table 2. Further, proteins with repeats tend to interact with more partners in protein–protein interaction networks [18] (Figure S7). With increasing complexity of an organism, the coordination of all genes and gene products needs to be more sophisticated. Many of the hubs in the eukaryotic interaction networks contain long domain repeats, possibly enabling more advanced cellular processes. This property of the domain repeats may explain why they are more abundant in the eukaryotes with larger proteomes.

**Table 2:**
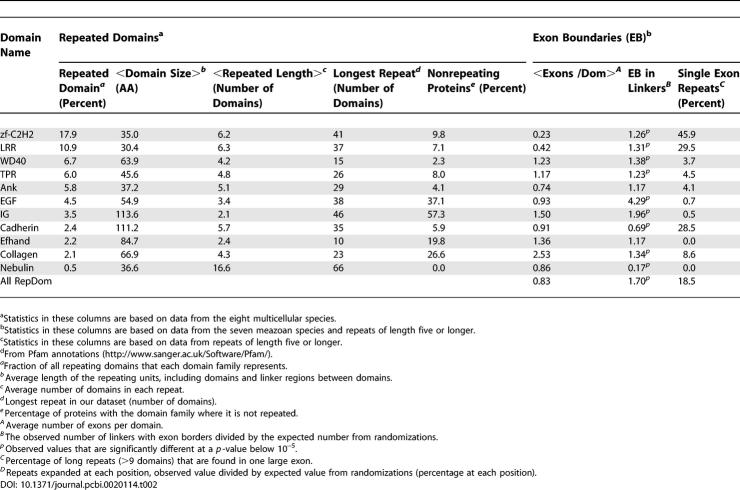
Repeat Statistics for the Domain Families

**Table 2:**
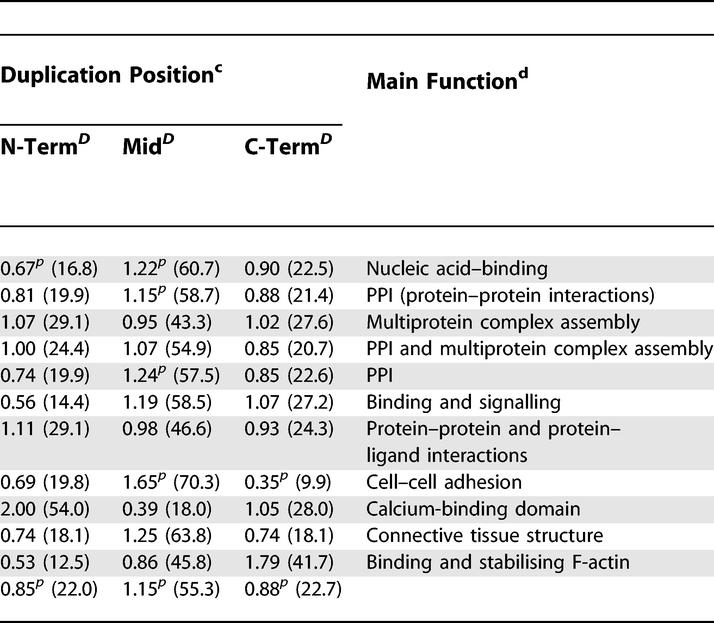
Extended

### Rapid Expansion of Repeated Domains

The repeated domains are more abundant than nonrepeated domains. In fact, nearly half of the assigned domains in the vertebrates are found in repeats (Table 1). Still, only 14% of all Pfam families form repeats. Furthermore, the ten largest domain families correspond to 62% of all repeating domains and are found in 48% of the proteins with repeats (Table 2). Hence, a few repeated domain families with high copy numbers account for a large portion of all domains (see Figure S1 for details, Protocol S1). This abundance can partly be explained by internal duplications, and, in addition, frequent duplications of the repeat-containing genes have been observed [19].

Further evidence of the frequent duplication in repeats is that orthologs appear to have expanded independently [12]. We found several such examples, one being the abnormal spindle-like microcephaly-associated proteins that in human consist of 71 IQ calmodulin–binding motifs. The protein has orthologs in other metazoans with fewer repeated domains, e.g., mouse (62 repeated domains), rat (62), zebrafish (63), chicken (53), and fruit fly (22). In worm, however, the longest repeat of this domain contains only six domains. Hence, it is likely that the repeat has been expanded independently in fruit fly and the chordates, or, alternatively, has been lost in C. elegans. In addition, further expansions may have taken place after the splits between fish, birds, and mammals, since the chicken proteins contain fewer repeated domains than the zebrafish ortholog.

Expansion of repeats through internal duplication is not unique to eukaryotes since some prokaryote-specific repeats can be found, e.g., the bacterial immunoglobulin (IG)–like domain and haemaglutinin repeats. Other prokaryotic repeats may be explained by horizontal transfer [19]. For instance, a 19-domain repeat of ankyrin domains is found in the syphilis bacteria Treponema pallidium. This domain family is found in other bacteria, but never with more than five consecutive domains, while in metazoa, the domain family is commonly repeated. Hence, a likely scenario is that this repeat has been horizontally transferred from a eukaryotic host, rather than expanded in the bacteria.

### Sequence Similarity Reveals Duplication Patterns

**Figure 3:**
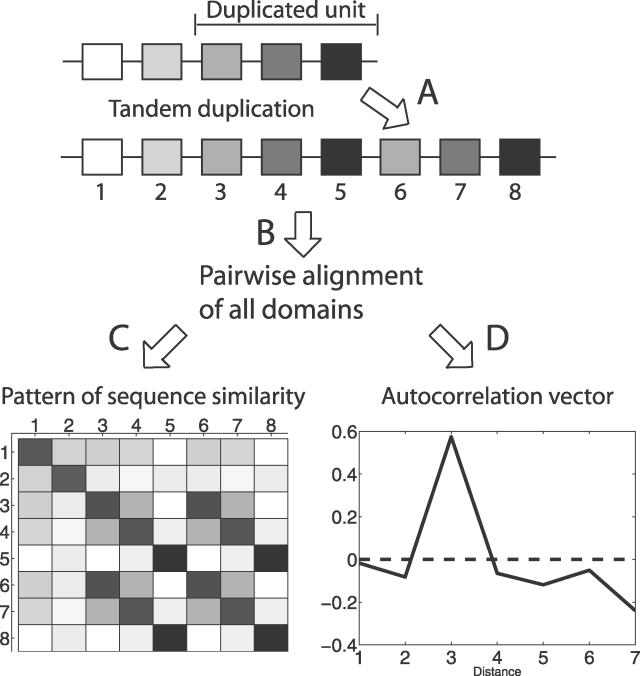
Overview of the Methodology (A) In a protein with five domains, a unit of three N-terminal domains has been duplicated in tandem. (B) To identify this evolutionary event, alignment of all domain pairs in the protein is performed. (C) The alignment scores between the domains displayed in a matrix with increasing color intensity for higher scores. The diagonal shows alignment scores for each domain to itself, while square 1,2 gives the score between the first and the second domain. A pattern where domain pairs 3–6, 4–7, and 5–8 have the highest alignment scores can be seen. (D) From the alignment scores, an ACV is calculated as the mean alignment score at each distance normalized around zero. The distance between the domains is defined as one for neighbouring domains, while domain pairs with one domain between them have distance two, etc. In this example a peak at distance three can be seen. Hence, we assume that this protein has evolved through the duplication of three domains.

The formation of repeats is not well-understood, therefore we aim to understand some of the underlying mechanisms of repeat expansion by studying the number of domains that is duplicated each time. Since domain repeats are assumed to be created through internal duplications [5], sequence similarity may provide information about recent duplications. Consequently, the pairwise sequence similarities between all repeating domains in a protein were examined using Smith-Waterman alignments [20]. The main outline of our methodology, as demonstrated in Figure 3, is to identify patterns of duplication from the alignments. To avoid bias towards duplications of few domains, only proteins with ten or more repeated domains were included.

**Figure 4:**
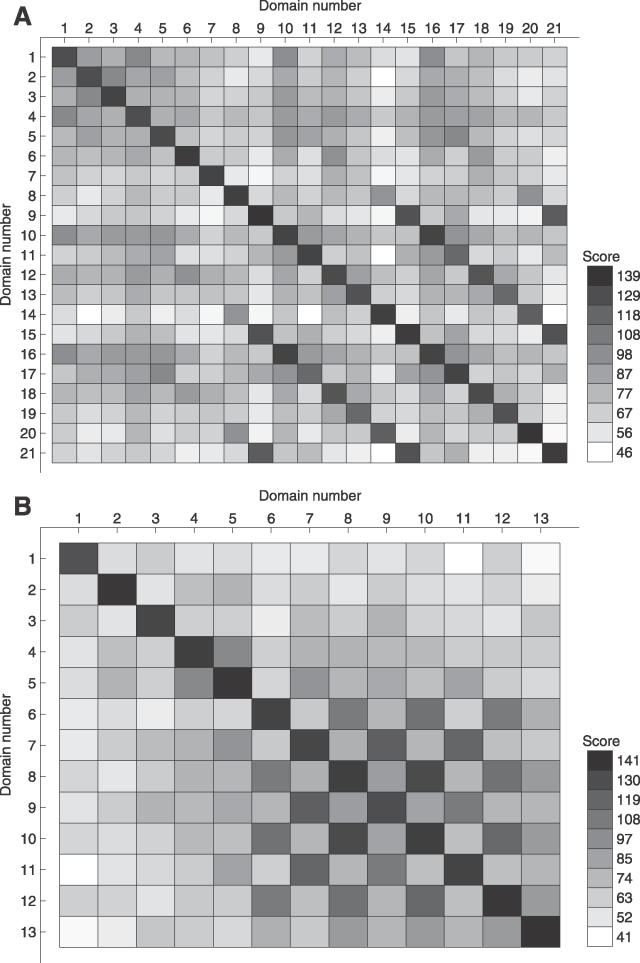
Pattern of Internal Domain Duplications in Two Human Proteins, ENSP00000319007 and ENSP00000303696, both with C2H2 Zinc Finger Repeats (A) ENSP00000319007. (B) ENSP00000303696. The intensity of the squares reflects the alignment score with darker color for higher scores. The numbers at each axis indicate the domains in N-to-C terminal orientation within the repeat. In these two examples, patterns of duplication of six domains (A) and two domains (B) can be seen.

Distinct patterns of repetition could often be distinguished, and in many proteins, units containing multiple domains have been duplicated in tandem. For instance, in the human zinc finger protein found in Figure 4A, it appears that a unit of six C2H2 zinc finger domains has been duplicated towards the end of the protein since domains at distance six (with five domains between them) have the highest sequence similarity. In another human C2H2 zinc finger repeat, though, a unit of two domains has been duplicated several times (Figure 4B). We noted that the size of the duplicated unit varied greatly with duplication of a single domain up to as many as nine domains at a time.

For many proteins, however, no clear pattern was seen since all domain pairs had similar alignment scores. In other proteins, there were mixed patterns within the protein as distinct parts of the protein have been expanded with duplication units of different sizes. Therefore, autocorrelation vectors (ACVs) were used to get a general view of the relative frequency of duplication units of different sizes in each protein. We have defined ACV as the average alignment score between domains at each distance, i.e., the alignment score between neighboring domains, domains at distance two, three, etc. (Figure 3). The peaks in such a vector should correspond to the most common sizes of duplication units in the evolution of the protein.

**Figure 5:**
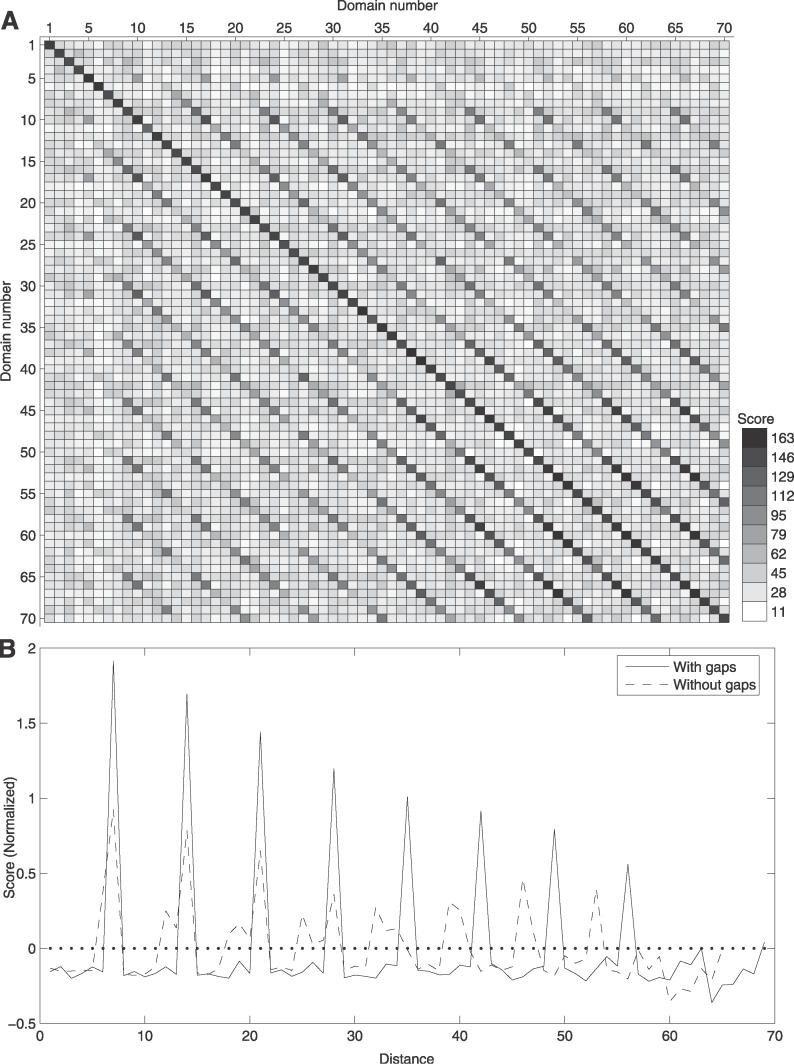
Pattern of Internal Domain Duplications in the Chicken Protein ENSGALP00000020382, with 66 Repeating Nebulin Domains (Pfam) (A) The intensity of the squares is related to alignment scores, and the numbers on both axes indicate the domains in N-to-C terminal orientation. As there were gaps in the repeat sequence (Figure 1), these were introduced as domains at positions 6, 18, 25, and 32. (B) ACV calculated from the alignment scores in (A) with the average similarity to domains at distance 1, 2, 3, etc. The ACV are normalized around zero, hence the dotted line at zero is the mean score between all domains in the protein. The ACV was calculated before introducing the gaps as domains (dashed line) and after (solid line). When the regions with no domain assignments were regarded as domains, the pattern of seven repeating units became much clearer, indicating that the gaps are also domains.

**Figure 6:**
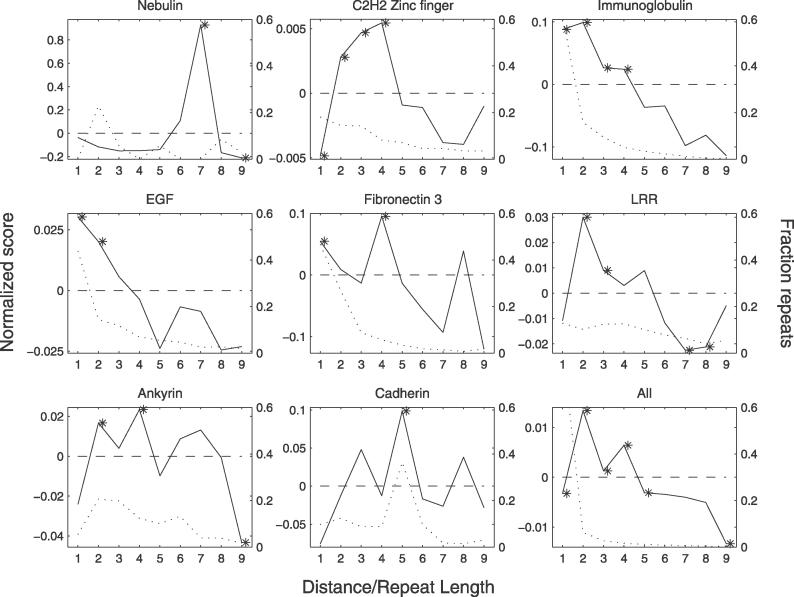
ACVs for Proteins with Repeats of Eight Different Domain Families Solid line shows ACVs for proteins with repeats of eight different domain families. In the bottom right diagram, the ACV for all proteins with repeats is displayed. The ACV for each family was normalized around zero, hence the dashed line at zero is the mean bit score between all domains in the family. The *p*-value for each datapoint was calculated from random shuffling of domains, and peaks with *p*-values below 10^−5^ are indicated with an asterisk. The dotted line illustrates the fraction of repeats of the domain family with each repeat length, i.e., nonrepeated proteins have length one. The number of proteins/domains that goes into each figure can be found in Materials and Methods. Data for the remaining domain families can be found in Figure S2.

The most common duplication pattern for a domain family can be elucidated when the average ACV for all repeats containing the family is calculated. As an example, the chicken nebulin protein (Figure 5) has been duplicated with seven domains at a time, and similar patterns were seen in most nebulin proteins. As a result, the ACV for all nebulin proteins show a clear peak at seven (Figure 6), indicating that duplication of a unit containing seven domains is dominant in the evolution of nebulin proteins.

Such clear patterns could not be found for all domain families, as can be seen in Figures 6 and S2. The C2H2 zinc fingers appear to be mainly expanded with two, three, or four domains, while duplication of one domain at a time is rare. A similar pattern is also seen for the ankyrin domains even if duplication of four domains is more dominant. The IG domains and the epidermal growth factor (EGF) domains, on the other hand, often show the highest similarity to the neighboring domain, and the similarity then decreases with distance. Hence, duplication of one domain at a time is the most likely scenario for their expansion. In addition, weak peaks at multiples of two can be seen for the IG family, indicating that this family also may expand by units of two domains. The fibronectin 3 domains are mostly expanded with a duplication unit of four domains, while LRR peaks at two and cadherin at five. Finally, when an ACV for all proteins with repeats was calculated, a duplication unit of two domains appeared to be most common for repeat expansion in general.

**Figure 7:**
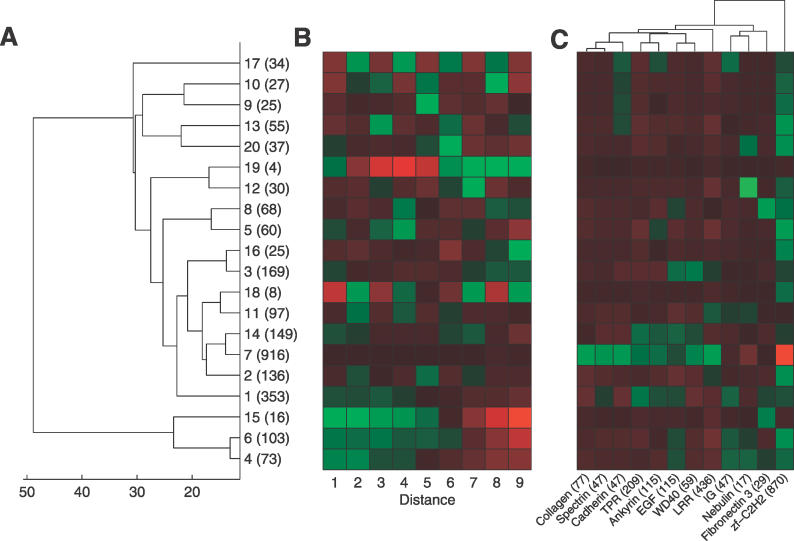
Hierarchical Clustering of the ACVs from Each Protein (A) Dendrogram of the 20 clusters. Each cluster is indicated by a cluster number followed by the number of proteins in the cluster. (B) The average ACV for each cluster with red color for values below the average and green for values above. (C) Distribution of the ten largest domain families, as well as nebulin, in the different clusters. The expected number of proteins from a domain family in each cluster was calculated using random shuffling, and Z-scores for overrepresentation (green) and underrepresentation (red) in the cluster were calculated. The numbers after the domain family names is the number of repeats of the family.

**Figure 8:**
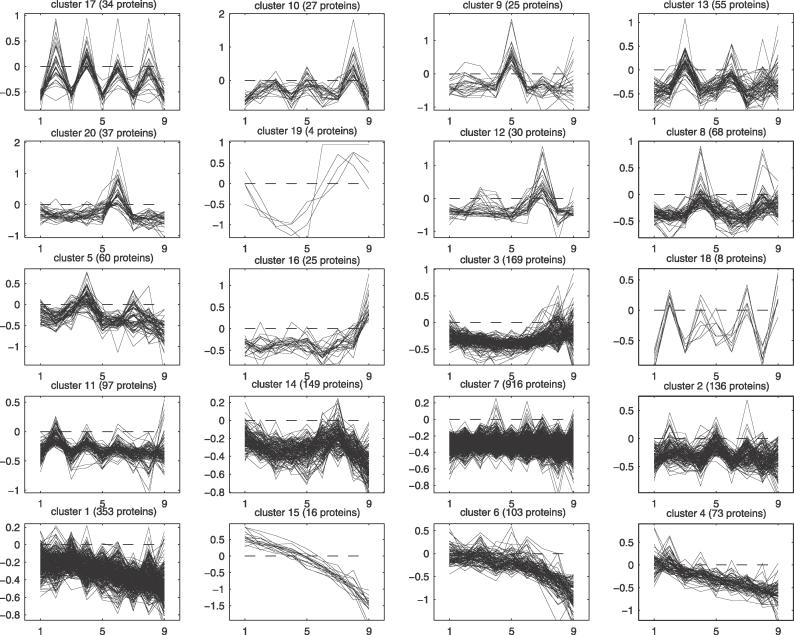
ACVs for All Proteins in Each of the 20 Clusters in Figure 7 The number of proteins in each cluster is indicated after the cluster number.

The ACVs show that duplication units of a few different sizes are dominant in each family. However, duplications of many different unit sizes may occur within a family. To get a view of how the patterns are distributed among the domain families, hierarchical clustering of the ACVs from all proteins was performed (Figure 7). Proteins with similar alignment scores between all the domains are clustered together in a few large clusters. These large clusters have a relatively “flat” ACV with no clear peaks at any distance (Figures 7B and 8). The distribution of the domain families in the different clusters is found in Figure 7C. As may be expected, most of the nebulin proteins are found in the same cluster (cluster 12), with a peak in the ACV at 7. Further, the C2H2 zinc finger proteins are evenly distributed in nearly all of the clusters except the largest cluster (cluster 7), where they are strongly underrepresented. In this large cluster, with repeats that have low sequence similarity among all the domains, we find representatives from most of the domain families, and especially collagen, spectrin, cadherin, and LRR. We speculated above that the IG repeats are either expanded by duplication of one or two domains. This assumption is further supported by the clustering of IG proteins in clusters with decreasing ACVs or peaks at multiples of two (clusters 1, 4, 6, and 11).

In conclusion, the domain repeats are most often created from the duplication of several domains at a time, while duplication of one domain appears to be less common. Further, the number of domains involved in each duplication event differs considerably within the domain families. However, for some domain families, there may be selection for duplication of a certain number of domains due to some functional or structural constraint, as is likely in the case of the nebulin domain. In addition, the most commonly repeated domains, the C2H2 zinc fingers, show the most diverse distribution of duplication patterns.

### Repeats Often Expand in the Middle

To determine if duplication at either end of a protein is preferred, the most recent duplications were identified and their positions were determined, revealing that a large proportion of the repeats have been expanded in the middle of the protein. The fraction of duplications we observe in the middle is slightly, but significantly, higher than expected by chance (Table 2). In addition, we found that additional domains from other families, which are not part of the repeat, did not have an effect on where the most recent duplications are located. Frequent duplications in the middle of a protein is in strong contrast to our recent findings that other multidomain proteins mainly evolve by the addition of a single domain at either termini [3,4]. Other types of domain shuffling may be constrained to the termini as additions of domains in the middle are likely to disrupt the tertiary/quaternary structure. However, duplication in the middle of a repeat does not necessarily affect the stability of the protein [21]. For most of the domain families, a similar distribution of duplication positions was found even if a few families differed. The nebulin domains, for instance, had a stronger bias for the C-terminal, while the thrombospondin type 3 repeats were mostly expanded at the N-terminal.

### What Determines the Duplication Sizes?

Repeated domain families are on average shorter than nonrepeated domain families [3]. However, we found no correlation between the size of a domain and the number of domains in each repeat (unpublished data). Instead, the number of domains in a duplicated unit was compared with the domain size. It could be expected that small domains are more often duplicated by many domains in tandem, while larger domains are duplicated one at a time. However, no correlation was seen between the number of duplicated domains and the domain size, measured as both number of amino acids and number of nucleotides (Figure S5). Hence, the mechanism that creates domain repeats is not likely to be dependent on the size of the duplicated region.

Another possibility is that there is a preference for duplication of certain sizes due to functional constraints, where a fixed number of domains are required for function. In that case, short repeats with that particular length may also be common. This seems to be true for cadherin domains, which have a peak in the ACV at distance five and are also abundant in five domain repeats (Figures 6 and S3). Further, many of the domain families with decreasing ACVs are commonly found as single domains, such as TPR, EGF, and IG. Still, the ACVs of all domain families cannot be explained by a preferred repeat unit size, e.g., the C2H2-zinc fingers are often found as single domains, even though duplication of one domain is rare according to the ACV.

### Exon Shuffling and Repeat Expansion

Exon shuffling, i.e., nonhomologous recombination in the intron regions, can create new exon combinations and new proteins. As a consequence, exon shuffling is responsible for many new domain combinations, and it has been demonstrated that exon-bordering domains often combine with other domains [22]. However, it is unclear if exon shuffling is also responsible for repeat expansion. In many instances, the repeated domains are spread over several exons, e.g., the collagen domain has on average more than two exons per domain.

To verify if the exon junctions are enriched in repeated domains or in linkers between the domains, simulations with random positioning of the junctions were performed. As a result, it was evident that more exon junctions are located in linkers than is expected at random (Table 2). Further, the enrichment in linkers is highly significant for some domain families, especially EGF and IG domains. Interestingly, IG and EGF are also the domain families that are most often found as single domains (Table 2) and the ones most often duplicated with one domain at a time (Figure 6). This could imply a mechanistic difference, where repeats expanded by exon shuffling are restricted to duplications of a single domain, while other duplication mechanisms are more likely to involve several domains. However, these two families do not constitute a large enough sample to draw general conclusions.

Our results are consistent with findings that extracellular domains, such as IG and EGF, are often recombined through exon shuffling [23]. However, the extracellular domain cadherin has significantly fewer linkers with exon junctions than expected. Another family where exon junctions are clearly underrepresented in the linker regions is the nebulin family. The nebulin protein in Figure 1 was examined, and the exon structure revealed the same exon pattern for each block of seven domains. If this duplication of seven domains should be regarded as exon shuffling, where four exons have been duplicated several times, or as another type of tandem duplication, is not evident, as each duplication could have occurred either within an exon or within a domain.

Interestingly, the exon structures revealed that 30% of the repeats with ten or more domains are located within one large exon, excluding the possibility of exon shuffling as the mechanism for their expansion. This was especially evident for human C2H2 zinc finger proteins, where 78% of the long repeats were found within one exon. The corresponding number of one-exon zinc finger repeats was lower in the other species, e.g., 11% in zebrafish. Also, LRR had many repeats in one exon, while other domain families always have the repeats spread over several exons (Table 2). Nevertheless, these large exons may be a consequence of intron loss, which would be more probable if the exons are old. We found, however, that the mean alignment score between domains in single-exon zinc finger repeats is slightly higher than for repeats that are distributed on several exons (alignment scores 74 ± 12 and 62 ± 21). Thus, they are more likely to be recently duplicated repeats. It is possible that duplication within an exon is more permissive as there are fewer problems with conservation of splice signals. Such duplications within an exon could in part explain the extensive duplication of zinc finger repeats in mammals. In addition, repeat expansion takes place in prokaryotes. Since they have no introns, exon shuffling cannot explain prokaryotic repeat duplications.

In conclusion, exon shuffling may be responsible for the expansion of some domain repeats, especially the extracellular ones that are often expanded one domain at a time. However, all repeat duplications cannot have been created by exon shuffling.

### Final Discussion

A complication in this analysis is deletions within proteins, since our method does not detect domain deletions. However, protein evolution tends to generate longer proteins, and it has been shown that proteins are more often extended by fusion than truncated by fission in protein evolution [24,25]. Further, it is likely that duplications are more common than deletions in repeat regions since the repeats have expanded so rapidly. Hence, we do not believe that deletions will affect our data to a large extent. Another problem is that some domains may be missed in the assignment process. Even though extended domain assignments were used, some domains are not detected, as demonstrated for the chicken nebulin protein in Figures 1 and 5. Still, we believe that good enough coverage of the repeats has been achieved for drawing general conclusions about the most common repeat expansion patterns.

Wright and coworkers recently published a study on protein aggregation where they found that neighboring domains, in repeats of IG and fibronectin domains, have lower sequence identity compared with more distant domains, and suggest that this may prevent protein aggregation [26]. For IG repeats, however, we found high sequence similarity for neighboring domains that decreases with distance (Figure 6), in contrast with the data presented by Wright et al. These differences are a consequence of different domain definitions, datasets, and methods to measure similarity (discussed in Table S2). We obtain lower similarities for neighboring domains in other domain families, such as C2H2 zinc fingers and ankyrins (Figure 6). These patterns may be a consequence of selection against aggregation. We believe, however, that duplication of several domains is the main contributor to this trend since the distribution of duplication patterns is quite broad. Nevertheless, selection against aggregation may favor duplications of several domains.

Whether repeat expansion is a random process or a controlled mechanism, where specific segments are selectively duplicated, remains to be discovered. Internal duplications may take place in all proteins, but it is likely that such duplications are lost if the protein does not contain domains that have a repeat-forming characteristic. On the other hand, an increase in the number of repeated domains might not alter the protein structure drastically and can actually promote protein stability [21,27]. The rapid expansion of repeats in eukaryotes and the duplications of identical segments several times in tandem suggest that a specific mechanism for their expansion could exist. Such a mechanism may involve a control on the DNA level that results in several duplications of the same segment.

Short protein repeats may be created from DNA hairpin formation and strand slippage while the hypermutability of minisatellite loci (repeating units of more than ten nucleotides) is thought to be due to recombination events [9]. The expansion of domain repeats may occur by a similar mechanism as the duplication of minisatellite loci, which have recombination hotspots that flank the duplicated regions [11]. If such recombination motifs are located in introns, the duplications would be regarded as exon shuffling. We also found that repeat expansion may, to some degree, work through exon shuffling. However, exon shuffling does not explain the evolution of all domain repeats, as many repeats are found within one large exon. Hence, if such motifs exist, they are located in the exons for some domain families, while in other families they are mainly found in the introns.

Identification of such hotspots would require exact identification of the gene segments that have been duplicated, which is difficult in most cases. Further, a method that would distinguish overrepresented DNA motifs at their flanks is needed. Finally, detection of such motifs would require that the motifs are conserved after the duplication has occurred. Still, many challenges lie ahead before the tandem duplication of protein domains can be fully understood.

### Conclusions

In this work, we show that repeat regions are most often created from the duplication of several domains at a time while duplication of one domain is less common. Further, we found that the internal duplications often occur in the middle of the repeats. Hence, the internal duplications in repeats evolve differently from other domain recombinations, which mainly involve the addition of a single domain at either terminus. Preference for duplication of a certain number of domains could be seen for some of the domain families. However, most domain families show broad distribution of duplication patterns and can be expanded with different numbers of domains, even if certain duplication sizes are more common. The exact mechanism behind these duplications is not well-understood. We found no correlation between the size of each duplicated fragment and the domain sizes. For some domain families, however, selection for functional units containing a certain number of domains may favor the duplication of that unit. In addition, exon shuffling could partly explain the duplications of some domain families, especially the extracellular domains. However, many repeats are found within one large exon, hence it is highly unlikely that they have evolved via exon shuffling.

## Materials and Methods

### 

#### Data.

We have analyzed the proteomes of 24 species; ten eukaryotes: *Homo sapiens, Mus musculus, Rattus norvegicus, Gallus gallus, Danio rerio, Drosophila melanogaster, Caenorhabditis elegans, Arabidopsis thaliana, Saccharomyces cerevisiae,* and *Schizosaccharomyces pombe;* seven bacteria: Escherichia coli K12, *Pseudomonas aeruginosa, Bacillus subtilis, Rickettsia conorii, Mycoplasma pulmonis, Prochlorococcus marinus,* and *Treponema pallidum;* and seven archaea: *Aeropyrum pernix, Methanococcus jannaschii, Nanoarchaeum equitans, Pyrococcus abyssi, Thermoplasma volcanium, Archaeoglobus fulgidus,* and *Methanosarcina mazei.*

The microbial sequences have been collected from the National Center for Biotechnology Information (NCBI) (ftp://ftp.ncbi.nlm.nih.gov/genbank/genomes/Bacteria/) and the eukaryotic genomes from Ensembl (ftp://ftp.ensembl.org/), except for S. cerevisiae and *S. pombe,* which were collected from *Saccharomyces* Genome Database [28] (http://yeastgenome.org/). In the eukaryotic species, the longest transcript from each gene was used.

Exon and intron information for the seven metazoan species were extracted from Ensembl (ftp://ftp.ensembl.org/).

#### Assignment of domains in repeat regions.

Pfam-A domains were assigned to the prokaryotic proteomes, the yeast species, and A. thaliana using HMMER (http://hmmer.wustl.edu) with a cutoff for assignments at an E-value of 0.1. This rather high cutoff was used to increase the number of assigned repeating domains. As we in this study focused on repeating domains (i.e., two or more identical domains found below the cutoff), the number of false positives is effectively reduced compared with when single domains are considered. After domain assignments, gaps within the repeats were evident in many cases, as shown in Figure 1. In many of those gaps, HMMER detected a domain from the same family, but with an E-value above the cutoff. As these are likely to be members of the same domain family, but have diverged too far to be detected at low E-values, all domains with adjacent assignments from the same family were also regarded as hits. The domain assignments for the remaining eukaryotic species were downloaded from the Ensembl database (ftp://ftp.ensembl.org/). When gaps of the same size or larger than the surrounding domains were found in the repeat regions, the proteins were subjected to additional assignments with HMMER as described above. All these additional assignments increased the number of domains in long repeats quite drastically, with additional domains in 40% of the proteins with more than ten repeating domains.

In addition, many repeats with alternating domains from related domain families were found, e.g., the different Pfam families of TPR or the IG-like domains. Such related domains are grouped together in the Pfam Clans (ftp://ftp.sanger.ac.uk/pub/databases/Pfam/). For a better view of repeat expansions, all families from the same clan were grouped together, rendering a slight increase in the number of repeated proteins, from 12.2% to 12.6%, and also an increase in the number of domains in each repeat, especially for proteins with LRR.

Throughout this study, a protein is regarded as a repeat-protein if it has at least two adjacent domains from the same family and no more than 100 unassigned residues between the domains.

#### Sequence comparisons and autocorrelation.

Analysis of evolutionary patterns was performed on proteins with repeat length ten or more, i.e., at least ten domains in tandem. The sequences of the repeating domains were extracted and aligned to each other using the Smith-Waterman alignment tool in the EMBOSS package [29] and default parameters. This gave pairwise alignment scores between all the individual domains in a repeat (Figure 3).

Our analysis is based on the assumption that the most recently duplicated domains have the highest sequence similarity to their originating domains. To quantify the duplication patterns, an *(ACV)* was calculated, i.e., the average alignment score between all domains at each distance *k,* where the distance between domain one and two is *k = 1,* between one and three is *k = 2,* and so on. If the alignment score between domains *d_i_* and *d_j_* is defined as *S(d_i,_d_j_)*, *ACV* is calculated as:

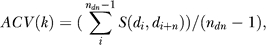
where *n_dn_* is the number of domain pairs with distance *k*. Finally, the vectors were normalized around zero so that *normACV* = *ACV/mean*( S̄). When the autocorrelation for all the repeating proteins with the same domain family was calculated, the average alignment score for all domains at distance *n* in all proteins with that family were used to calculate *ACV(k)*. Before these calculations, the dataset was homology-reduced using nrdb90 [30], removing all sequences with identical domain architectures and more than 90% sequence identity.

The ACVs presented in Figure 6 contain the following numbers of proteins and domains: Nebulin (nP_A_ = 17 (number of proteins for ACV calculation), nD_A_ = 460 (number of domains for ACV), nP_s_ = 16 (number of short repeats, length < 10)); C2H2 Zinc finger (nP_A_ = 870, nD_A_ = 13,244, nP_s_ = 3,043); IG (nP_A_ = 47, nD_A_ = 850, nP_s_ = 3,002); EGF (nP_A_ = 115, nD_A_ = 1,844, nP_s_ = 1,818); Fibronectin 3 (nP_A_ = 29, nD_A_ = 419, nP_s_ = 1,160); LRR (nP_A_ = 436, nD_A_ = 6,730, nP_s_ = 1,856); Ankyrin (nP_A_ = 115, nD_A_ = 1,793, nP_s_ = 1,373); Cadherin (nP_A_ = 47, nD_A_ = 972, nP_s_ = 504); All (nP_A_ = 2,386, nD_A_ = 36,926, nP_s_ = 211,403).

#### Clustering of ACVs.

ACVs of length nine were created for all proteins with ten or more repeats. As longer vectors cannot be created for proteins with repeat length ten, we used this cutoff to be able to compare the whole dataset. Hierarchical clustering of the ACVs was performed using the Ward incremental sum of squares distance measure, in Matlab (The MathWorks, Natick, Massachusetts, United States), to measure similarity between the vectors. The distance is defined as

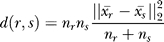
where n_r_ and n_s_ are the number of objects in clusters *r* and *s,* and 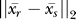 is the centroid distance between the clusters. The clustering was stopped at 20 clusters, as too many clusters are difficult to visualize, while fewer clusters only increased the number of members in the largest cluster when the smaller clusters were removed.

In addition, the domain families were also clustered with the same method using the distribution of domain families in the 20 ACV clusters.

#### Position of latest duplication.

The position of latest duplication was determined for all proteins with repeats of five or more domains. To identify where in the repeat the most recent duplication took place, a matrix was created similar to the one in Figure 4. Alignment scores that were more than one standard deviation over the mean alignment score in the protein were identified as possible latest duplications, and their values were set to one. All other values were set to zero, giving a matrix where only significantly high alignment scores have values. Then, the longest diagonal with ones in the matrix was regarded as the latest duplication event. If several diagonals with the same length were found, the one with the highest alignment scores was selected. Finally, the position of the latest duplicated diagonal was determined as N-terminal, C-terminal, or middle. Alternative methods to evaluate the position of latest duplication have been evaluated. These are described in Table S1, and this method is also described in further detail in Figure S4.

#### Statistical tests.

To estimate the statistical significance of our results, Z-scores were calculated from randomization in 10,000 iterations. The Z-score was calculated as *Z* = |*x* − *μ*|/*σ*, where *x* is the observed value and *μ* is the average value obtained from simulations with standard deviation *σ.* Assuming a normal distribution of the data, the *p*-value was then derived from the Z-score using normal distribution *p*-value tables.

In the simulation of ACVs, the positions of the domains in a protein were shuffled while maintaining their individual alignment scores. In each iteration, an ACV for proteins with each domain family was calculated, and finally the Z-score for each position of the vector was calculated from these randomized values.

In the case of enrichment of exon boundaries in linker regions, the domain and linker positions in each protein were kept constant. The number of exon boundaries in each protein was also conserved, but they were positioned randomly along the protein sequence. In each iteration, the fraction of linkers that contained exon boundaries was calculated.

The enrichment of the domain families in each cluster in Figure 7 was calculated by randomly placing the proteins in different clusters while maintaining the number of proteins in each cluster. Then the observed number of proteins from each family, in each cluster, was compared with the values from randomization and Z-scores for underrepresentation or overrepresentation were calculated.

For estimation of the position of latest duplication, the domain order was shuffled in each protein while maintaining individual alignment scores. In each iteration, the fraction at each position was estimated, as described in the previous section. Finally, the Z-scores for fraction at N/C-terminal or middle were calculated.

## Supporting Information

Figure S1 Distribution of Domain Family Copy Number of Human Domains Where Repeating Families and Nonrepeating Families Have Been Separated A repeated domain family is defined as a family found in a repeat of at least three domains, and nonrepeated families are never found as repeated. The copy numbers for repeated domains have been calculated as the total number of copies (Rep. Copies) or counting each protein with the repeat only once (Rep. Compressed).
(24 KB EPS)

Figure S2 ACVs for the Different Domain Families The domain family name is followed by the number of proteins (nP) and number of domains (nD) used in the calculations. The autocorrelation for each family was normalized around zero, hence the dashed line at zero is the mean bit score between all domains in the family. The *p*-value for each datapoint was calculated from random shuffling of domains, and peaks with *p*-values below 10^−5^ are indicated with an asterisk.
(61 KB EPS)

Figure S3 Fraction of Domain Repeats (with Nine or Fewer Domains) That Has Repeat Length 1, 2, 3, etc., Calculated for Each of the Domain Families in Figure 2
(47 KB EPS)

Figure S4 Determining the Position of Latest Event (A) The alignment scores between all domains in a human zinc finger protein with darker color for higher scores.
(B) All scores that are one standard deviation over the mean score are set to one (gray). Then the longest diagonal of “ones” is identified (black) and the position of that diagonal is determined. In this case the latest duplication is estimated to occur in the end.
(168 KB EPS)

Figure S5 Secondary Structure of the Repeated Regions and Other Regions of the Proteins The fraction of different regions that contain disordered regions or different secondary structures. The first bar shows the distribution in all of the proteins followed by repeated domains (RepDom), non-repeated domains (NRDom), and regions without domain assignments (Unass).
(25 KB EPS)

Figure S6 For Each Domain Family, the Number of Domains in Most Duplicated Units Is Compared with the Mean Domain Sizes The size of most duplicated units, i.e., the number of domains involved in most duplications, was determined from the highest peak in the ACVs (Figure 5) for the 34 largest repeating domain families. Theses values are compared with the average size of a domain in (A) amino acid residues and in (B) nucleotide base pairs.
(884 KB EPS)

Figure S7 The Average Number of Interaction Partners (Connectivity) in the IntAct Protein Interaction Networks, with Error Bars The connectivity is displayed for proteins with no repeat (repeatlength 1), two-domain repeats, etc., up to repeats of length nine or more. The networks for three eukaryotic species, *D. melanogaster, C. elegans,* and S. cerevisiae are displayed, and they all show higher connectivity with increasing repeat length.
(26 KB EPS)

Table S1 Predicted Position of Latest Duplication with Different Cutoffs for the Two Methods LD and 3P Using Repeats of Length 10 or More (21 KB DOC)

Table S2 Fraction of Domain Pairs with >30% Sequence Identity for Adjacent and Nonadjacent Domains of IG and Fn3 (28 KB DOC)

Protocol S1 Supplementary Material (60 KB DOC)

## Abbreviations

ACV: autocorrelation vectors
EGF: epidermal growth factor
IG: immunoglobulin
LRRs: leucine rich repeats
TPR: tetratrico peptide repeats
