# Supplementary material for: Expansion of Protein Domain Repeats
Source: PLoS Comput Biol. 2006 Aug 25;2(8):e114. doi: 10.1371/journal.pcbi.0020114 (PMC1553488; doi:10.1371/journal.pcbi.0020114)
Supplement: Table S1 — (21 KB DOC) [file pcbi.0020114.st001.doc]

**Table 1:** Predicted postition of latest duplication with different cutoffs for the two methods LD and 3P using repeats of length 10 or more.

| Method | Cutoff | C-terminal* | Middle* | N-terminal* | Proteins** |
| --- | --- | --- | --- | --- | --- |
| LD | 2 STD | 7.6*^p^* | 81.8 *^p^* | 10.6*^p^* | 2406 |
| LD | 1 STD | 10.6 *^p^* | 74.1 *^p^* | 15.3 | 2605 |
| LD | 1/2 STD | 14.3 *^p^* | 66.1 *^p^* | 19.6 | 2611 |
| LD | 0 STD | 18.8 *^p^* | 52.7 | 28.4 | 2614 |
| 3P | 0 STD | 23.5 | 42.3 | 34.26 | 2615 |
| 3P | 1 STD | 20.8 | 42.5 | 36.73 | 795 |

* Percentage of repeat expansions predicted as N/C-terminal or in the middle
** The number of proteins with values over the cutoff
*^p^* Observed values that are significantly different at a P-value below 10^-5^.
