# Supplementary material for: Expansion of Protein Domain Repeats
Source: PLoS Comput Biol. 2006 Aug 25;2(8):e114. doi: 10.1371/journal.pcbi.0020114 (PMC1553488; doi:10.1371/journal.pcbi.0020114)
Supplement: Table S2 — (28 KB DOC) [file pcbi.0020114.st002.doc]

**Table 2:** Fraction of domain pairs with >30% sequence identity for adjacent and non-adjacent domains of Ig and Fn3.

| Repeat length | Ig Adjacent | Ig Non-Adjacent | Fn3 Adjacent | Fn3 Non-Adjacent |
| --- | --- | --- | --- | --- |
| 2-5 | 37.8 | 47.4 | 37.0 | 45.9 |
| 6-9 | 42.2 | 42.4 | 57.3 | 47.1 |
| 10+ | 77.0 | 65.0 | 44.1 | 42.1 |

|  |
| --- |
