# Supplementary material for: Expansion of Protein Domain Repeats
Source: PLoS Comput Biol. 2006 Aug 25;2(8):e114. doi: 10.1371/journal.pcbi.0020114 (PMC1553488; doi:10.1371/journal.pcbi.0020114)
Supplement: Protocol S1 — (60 KB DOC) [file pcbi.0020114.sd001.doc]

# Supplementary material for: Expansion of protein domain repeats

**Åsa K. Björklund, Diana Ekman
and
Arne Elofsson** [*](#foot179)

* To whom correspondence should be addressed

Fax: +46-8-164672

Tel: +46-8-153679

Email: arne@bioinfo.se

Stockholm Bioinformatics Center,

Center for Biomembrane Research,

Stockholm University,

SE-10691 Stockholm,

Sweden

# Copy numbers of repeated domains

A well studied feature of all domain families is that they exhibit a power-law distribution where a few families exist in many copies while most have few copies [[1](../../../../arnee/textdoc/papers/repeats/supplement3/supplement3.html#Qian:2001)]. However, this power-law behavior is different for repeating and non-repeating domains. As can be seen in FigureS[1](#fig:powerlawCopies), a larger fraction of the repeating domains have high copy-numbers, giving a less steep power-law slope when only repeated domains are considered. The high copy numbers of repeated domains is partly a consequence of the difference in duplication mechanism compared to non-repeated domains. New copies of non-repeated domains should mainly originate from duplications of genes, while new repeating domains, in addition to gene duplications, can be created from internal tandem duplications [[2](../../../../arnee/textdoc/papers/repeats/supplement3/supplement3.html#Andrade:2001b)]. However, the main difference between repeated and non-repeated domains can be contributed to frequent duplication of repeat-containing genes. When each protein with repeated domains is counted as one copy, regardless of the number of repeated domains it contains, the power-law slope is still less steep than the slope for non-repeated domains.

# Autocorrelation vectors and domain units

The autocorrelation vectors (ACVs) for each of the domain families is calculated as described in the original manuscript. In Figure S[2](#fig:acv) the ACVs for the largest repeating domain families are provided. The calculations are based on proteins with at least ten repeated domains and only families that are found in at least five proteins with such long repeats are included. In these 30 domain families the main duplication patterns are quite varied where some show decreasing similarity with increasing distance while others have clear peaks at different positions.

One explanation for why the domain families have preferences for duplication of different sizes could be that there are functional units of certain sizes. If shorter repeats (<10 domains) are more often found in a certain size, this would indicate that a functional unit of that size is preferred. Figure S[3](#fig:short) displays the fraction of repeats of each length for all the domain families in Figure S[2](#fig:acv). For some domain families the prefferred duplication sizes, is well correlated with the most common domain size, especially Cadherin where a clear overrepresentation of five-domain repeats is found. Further, many of the domain families with decreasing ACVs are commonly found as single domains, such as TPR, EGF and IG. Still, the ACVs of all domain families cannot be explained by a preferred repeat unit size, e.g. the C2H2-zinc fingers are often found as single domains, even though duplication of one domain is rare according to the ACV.

# Position of latest duplication

The position of the latest duplication was estimated from the matrix of alignment scores, as found in Figure S[4](#fig:exPosition)A. The method we used to determine the position is based on finding the longest diagonal, hence we refer to it as ``Longest Diagonal'' (LD). Alignment score values that were more than one standard deviation over the mean alignment score between all domains in the protein were identified as possible latest duplications and their values were set to one. All other values were set to zero giving a matrix where only significantly high alignment scores have values (Figure S[4](#fig:exPosition)B).

Then, the longest diagonal with ones in the matrix was regarded as the latest duplication event (black in Figure S[4](#fig:exPosition)B). If several diagonals with the same length were found, the one with the highest alignment scores was selected. Finally the position of the latest duplicated diagonal was either determined as ``N-terminal'' if the first domain in the diagonal was most identical to domain one, i.e. if the diagonal runs out through the top of the matrix. Or as ``C-terminal'' if the last domain was most identical to the final domain in the protein as is the case in Figure S[4](#fig:exPosition) and else as ``Middle''.

As it is not trivial how to define the latest duplication event, and thereby determining its position, different methods for determining the position were evaluated. For instance, in the method above (LD) different cutoffs for significant alignment scores were tested, one of them being one standard deviation over the mean. Examples of results obtained with different cutoffs can be found in Table S[1](#tab:position).

Another method is based on dividing a repeat into three parts containing an equal number of domains. Then the highest alignment score for each of the domains was identified and, for each part, the mean of all highest scores was calculated. Subsequently, the part containing the highest average score was defined as the region where the latest duplication event had occurred, i.e. N/C-terminal or middle part. This method is referred to as ``three parts'' (3P) in the table below. As many proteins have similar scores in all three parts, a cutoff was applied to define if the difference in score was significant. This cutoff was based on the standard deviation of the highest scores, e.g. it was considered significant if the highest scoring part had a value more than one standard deviation over the values of the other two parts. Different cutoffs in relation to the standard deviation was tested, however they gave similar results, only the number of proteins were reduced with higher cutoffs. Some examples are provided in Table S[1](#tab:position).

All these cutoffs predict that most of the duplications have occurred in the middle of the proteins. In addition, a slight preference for duplications at the N-terminal can be seen with all methods. As we cannot determine what the best cutoff for predicting the position is, we can only draw conclusions about the general trend. In addition, there are more middle domains than terminal domains in a repeat. Hence, by chance more duplications should occur in the middle. This was confirmed through random shuffling of the domain positions which also gave more repeats in the middle. However, these randomizations indicate that we observe significantly higher frequencies of repeats in the middle than expected by chance.

# Adjacent/non-adjacent domains of Ig and Fn3

To investigate why we obtain different results compared to the study by Wright et al [[3](../../../../arnee/textdoc/papers/repeats/supplement3/supplement3.html#Wright:2005)], the two domain families immunoglobulin (Ig) and fibronectin 3 (Fn3) were studied in detail. Wright and coworkers showed that fewer adjacent domains have high sequence similarity (>30%) compared to non-adjacent domains for both of these families. Adjacent domains were defined as being less then 30 residues apart while non-adjacent domains are found in the same protein at longer distances or with other domains in between. However, using autocorrelation vectors, we found that both Ig and Fn3 domains have high sequence similarity if they are adjacent, except that Ig has a peak in the ACV at distance 2 and Fn3 at distance 4 (Figure S[2](#fig:acv)). However, we only used repeats with ten or more domains in our analysis and a different domain definition was used (Pfam domains instead of Scop superfamilies).

Therefore, alignments between all Ig an Fn repeats were performed, and the fraction of adjacent and non-adjacent domains that had sequence identity over 30% was calculated using the same criteria as Wright et al with a cutoff of 30 residues and repeats of all lengths. We found that for shorter repeats, two to five domains, the non-adjacent domains have higher sequence identities, while this is not the case for longer repeats (Table S[2](#tab:igfn)). As we have more long repeats in our dataset, due to extended assignments in repeat regions, it would be expected that we see a different trend.

# Secondary structure of repeats

The secondary structure of all the proteins in our dataset was determined using Psipred [[4](../../../../arnee/textdoc/papers/repeats/supplement3/supplement3.html#psipred)] and disordered regions using Disopred [[5](../../../../arnee/textdoc/papers/repeats/supplement3/supplement3.html#disopred)]. These results are shown in (Figure S[5](#fig:secstruct)).

It has been demonstrated that disordered regions are often involved in binding to other proteins [[6](../../../../arnee/textdoc/papers/repeats/supplement3/supplement3.html#Brown:2002),[5](../../../../arnee/textdoc/papers/repeats/supplement3/supplement3.html#disopred)]. However, we did not find an increase in disorder in repeated domains even if the repeating domains mainly have functions in binding to other proteins (Figure S[5](#fig:secstruct)). We only confirmed earlier findings [[7](../../../../arnee/textdoc/papers/repeats/supplement3/supplement3.html#Ekman:2005)] that regions with no domain assignments are enriched in disorder.

Investigation of the secondary structure revealed that a large fraction of the repeated domains consist of beta-sheets, while few alpha-helices were found (Figure S[5](#fig:secstruct)). This is not a surprising finding since many of the repeated domains consist of beta-sheets, e.g. immunoglobulin, EGF, WD40 and LRR. However, some families are mainly alpha-helical, e.g. TPR, collagen and efhand. Still, all these domain families form repeats that mediate similar functions such as protein-protein interactions.

# Size of duplicated unit and domain size

Analysis of the size of a domain and the number of domains in each duplicated unit was performed to see if there is any correlation between them. The most common size of a duplicated unit was determined for each domain family from the ACV plots (Figure S[2](#fig:acv)), where the highest peaks would correspond to the most common unit. The average size of the domain families was defined for each repeat unit (domain including linkers) both from the amino acid sequences and from the size of the genome each repeat unit covers. However, no correlation was seen between the number of duplicated domains and the domain size, neither at amino acid or nucleotide level (Figure S[6](#fig:domlen)). Hence, the mechanism that creates domain repeats is not likely to be dependent on the size of the duplicated region.

# Repeats in protein-protein interaction networks

As domain repeats are involved in interactions with other proteins, it could be expected that they have more interaction partners. This was confirmed for yeast proteins in another study by us [[8](../../../../arnee/textdoc/papers/repeats/supplement3/supplement3.html#Ekman:2006)]. Here, we have further investigated repeats in the three most extensively studied eukaryotic protein-protein interaction networks (PPINs) in the IntAct database [[9](../../../../arnee/textdoc/papers/repeats/supplement3/supplement3.html#intact)]. In all three networks, proteins with repeats tend to have more interaction partners than proteins with no repeats, and the connectivity increases with repeat length (Figure S[7](#fig:interaction)).

The protein-protein interaction data was collected from the IntAct database [[9](../../../../arnee/textdoc/papers/repeats/supplement3/supplement3.html#intact)] (release February 14th, 2006) and all interactions were collected without any filtering. The interaction networks used in this study was from D. melanogaster, C. elegans and S. cerevisiae. The three datasets contained respectively: 6636, 1911 and 4986 proteins. The fraction of proteins with repeats was higher than in the whole proteomes (Table 1 in original manuscript), with 8% in S. cerevisiae and 14% in D. melanogaster and C. elegans.

# Alignment of the Nebulin protein example

Alignment of the nebulin protein ENSGAL00000020382 in Figure 1 and 4 with domain number followed by a letter I for initial domain assignments, A for additional domain assignments or G for gaps. The numbers after the index give the position of the domain.

7I.227-255 DSPVQKQAEINSKQLSDKLYRSSGEEVKH-

14I.471-499 DSMTMVLAQHNTKQLSDVAYKQEGEKVKH-

35I.1203-1231 DAMDMVLAKHNADIMNKHAYTQAWEKDKT-

49A.1689-1717 DSMPMVLAKHNSEIMNHVSFLSAWEKDKT-

56I.1932-1960 DSLEMTLAKHNAEMMNKRLYTEAWDKDKT-

63I.2175-2203 DSLEMTLAKHNAETMNKRLYTEAWNKDKT-

70I.2418-2446 DSLAMMLAKHNAEIMNKRLYTEAWDADKT-

21I.715-743 DAMEQVLAKQNAKTMNKRLYTDKWNKEKT-

42I.1446-1474 DAMNIILAKSNAKNRSDILYREAWDKDKT-

28I.959-987 DSMNMALALHNAKIMDEHQYKQAWEEDKK-

8I.262-290 DVPQFIQARYNAANVSDAYYKQDYHDLIA-

15I.506-534 DVPQFIQARVNAFNLSDANYKADWKKTIA-

29I.994-1022 DIPQFALAKANAFNISDKMYRHSFEEARK-

43I.1481-1509 DTPEILLAKSNLINTSDKMYRLAMEEDKK-

50I.1724-1752 DTPGILLAQQNKVNYSEKMYRLAMEEDKK-

64I.2210-2238 DTPEILLAKQNQAHYSQKMYKLALEESKK-

57I.1967-1995 DTPEITLAKQNMHNYSEKLYKQAMEEAKK-

36I.1238-1266 DTPDILQAKQNKANYSQKQYKLDWQEMIK-

22I.750-778 DTPEILQSRVNQITMSNKLYKAGWEEDKK-

1I.14-42 PCYDVVIAKMNAENLSMKKYQEDFENVKD-

10I.336-364 DDPKLVHYMHVAKIQSDREYKKDYEKSKT-

17I.580-608 DDPKLVHYMHVAKMQSDREYKKDYEVTKT-

31I.1068-1096 DDPKLVHYMQVAKMQSDREYKKAYETSKT-

24I.824-852 DDPKLVHFMQVAKMQSDREYKKDYEKAKT-

52I.1798-1826 DDPKMMWSMHVAKIQSDREYKKAFEKTKT-

59I.2041-2069 DDPKMMWSMHVAKIQSDREYKKAFEKTKT-

66I.2284-2312 DDPKMMWSMHVAKIQSDREYKKAFEKTKT-

45I.1555-1583 DDPKMMWSMHVAKVQSDREYKKAFEKTKT-

38I.1312-1340 DDPKMMWSMQVAKMQSEREYKKDFEKWKT-

5I.159-187 EDPHQIHCMKVEAMKSDKNYKADYEEEKT-

2I.50-78 ETPEYEANKRVSDNVSKIKYRADYEKNKA-

3I.89-117 ENPLLRQLKTAGDVLSDKLYKEAYERSKG-

41A.1413-1441 GSLESEKNKKASEILSEKKYRQHPDTIKF-

48A.1656-1684 GSLDEEKNKRASMILSDKKYRQHPDTIKF-

13A.438-466 GSLDVEKAKKAGDALNEKKYRQHPDTIKF-

55A.1899-1927 GSLDIEKAKRAGQILSDKVYRQPPDTIKF-

62A.2142-2170 GSLDVEKAKRAGQILSDKVYRQPPDTIKF-

69I.2385-2413 GSLDVEKAKKAGEILSDRKYRQPADQIKF-

20I.682-710 QSLEVEKAKKASEILSEKKYRQHPDKLKY-

27A.926-954 GSLEAEKNKKAMEILSEKKYRQHPDKLKY-

34A.1170-1196 GSLDVEKSKKATEIASDQKYRQHPSIF---

9I.298-326 DAIPITRAKASRNIASDYKYKEAYEKAKG-

16I.542-570 DAIPIIAAKASRNIASDYKYKESYEKDKG-

44I.1517-1545 DAIPIKAAKASRDIASDYKYKEGYRKQLG-

51I.1760-1788 DAIPIKAAKASRDIASDYKYKEGYRKQLG-

58I.2003-2031 DAIPIQAAKASRQIASDYKYKEGYRKQLG-

65I.2246-2274 DAIPIQAAKASREIASDYKYKEGYRKQLG-

37I.1274-1302 EAISVKAAKASRDIASDYKYKEGYRKQQG-

30I.1030-1058 DAIPIKAAKASRDIASDYKYKLGYEQDKG-

23I.786-814 DAIPIKAAKTSQDIASDYKYKLAHEKAKG-

18GI.587-671 DMFSVTAAKKAQEAVTNTGYKQLIHHYTL-

25G.859-887 DMLSVVAAKKAQEVATNANYKNLIHVYNV-

32G.1104-1131 DALSIMAAKEAQDRVTNANYKRLIHHYML-

11A.371-395 DTFSIQAAKKSQDVASTAHYKNLIH-----

39A.1347-1375 DMLGFLLAKKCQELVSDIDYKHMLHRWTC-

46A.1590-1618 DMLGIVLAKKCQELVSDVDYKHLLHRWTC-

53A.1833-1861 DMLGIVLAKKCQELVSDVDYRHYLHQWIC-

60A.2076-2104 DMLGIVLAKKCQELVSDVDYRHYLHQWIC-

67A.2319-2347 DMLGIVLAKKCQELVSDVDYRHYLHQWIC-

12I.402-430 DAMDVELAKNMMQIQSDNVYKQDYNSWFK-

33I.1134-1162 DAMSFELYRNMNQIQSNNEYKQDYNEWFK-

26I.890-918 DAMSLELAKNMMQIQSNNQYRAEYDESMK-

19I.646-674 DSVNLELSRNMMQLQSDNMYKADFNNWLR-

54I.1864-1892 DQNDVIHARKAYDLQSDNFYKSDL-EWMRG

61I.2107-2135 DQNDVIHARKAYDLQSDNFYKSDL-EWMRG

68I.2350-2378 DQNDVIHARKAYDLQSDAVYKSDL-EWLKG

47I.1621-1649 DQNDVVQARKVYDLQSDNVYKSDL-QWLRG

40I.1378-1406 DQNDVTQAKRVYELQSDNLYKSDL-QWLKG

4A.124-151 -ETPKFQTDNALKNFSDVKYKDAYQKNIL-

6G.194-222 TITQEYEAIKKLEQCKDHTYKKHPDQIKF-

## Bibliography

1

Qian J, Luscombe NM, Gerstein M (2001) Protein family and fold occurrence in genomes: power-law behaviour and evolutionary model. J Mol Biol 313:673-681.

2

Andrade M, Perez-Iratxeta C, Ponting C (2001) Protein repeats: structures, functions, and evolution. J Struct Biol 134:117-131.

3

Wright C, Teichmann S, Clarke J, Dobson C (2005) The importance of sequence diversity in the aggregation and evolution of proteins. Nature 438:878-881.

4

Jones D (1999) Protein secondary structure prediction based on position-specific scoring matrices.
J Mol Biol 292:195-202.

5

Ward JJ, Sodhi JS, McGuffin LJ, Buxton BF, Jones DT (2004) Prediction and functional analysis of native disorder in proteins from the three kingdoms of life. J Mol Biol 337:635-645.

6

Brown C, Takayama S, Campen A, Vise P, Marshall T, et al. (2002) Evolutionary rate heterogeneity in proteins with long disordered regions. Mol Evol 55:104-110.

7

Ekman D, Björklund ÅK, Frey-Skött J, Elofsson A (2005) Multi-domain proteins in the three kingdoms of life - orphan domains and other unassigned regions. J Mol Biol 348:231-243.

8

Ekman D, Light S, Björklund ÅK, Elofsson A (2006) What properties characterize the hub proteins of the protein-protein interaction network of Saccharomyces cerevisiae? Genome Biol 7:R45.

9

Hermjakob H, Montecchi-Palazzi L, Lewington C, Mudali S, Kerrien S, et al. (2004) Intact: an open source molecular interaction database. Nucleic Acids Res 32:D452-5.

# 
